# The economic impact of anastomotic leakage after colorectal surgery: a systematic review

**DOI:** 10.1007/s10151-024-02932-4

**Published:** 2024-05-20

**Authors:** David J. Nijssen, Kiedo Wienholts, Maarten J. Postma, Jurriaan Tuynman, Willem A. Bemelman, Wytze Laméris, Roel Hompes

**Affiliations:** 1 Department of Surgery, Amsterdam UMC Location University of Amsterdam, Amsterdam, The Netherlands; 2 Treatment and Quality of Life, Cancer Center Amsterdam, Amsterdam, The Netherlands; 3 Imaging and Biomarkers, Cancer Center Amsterdam, Amsterdam, The Netherlands; 4 Department of Health Sciences, University Medical Center Groningen, University of Groningen, Groningen, The Netherlands; 5 Department of Economics, Econometrics and Finance, Faculty of Economics and Business, University of Groningen, Groningen, The Netherlands; 6 Department of Surgery, Amsterdam UMC Location Vrije Universiteit, Amsterdam, The Netherlands

**Keywords:** Anastomotic leakage, Socioeconomic burden, Health economic evaluation, Cost analysis, Systematic review

## Abstract

**Background:**

Anastomotic leakage (AL) remains a burdensome complication following colorectal surgery, with increased morbidity, oncological compromise, and mortality. AL may impose a substantial financial burden on hospitals and society due to extensive resource utilization. Estimated costs associated with AL are important when exploring preventive measures and treatment strategies. The purpose of this study was to systematically review the existing literature on (socio)economic costs associated with AL after colorectal surgery, appraise their quality, compare reported outcomes, and identify knowledge gaps.

**Methods:**

Health economic evaluations reporting costs related to AL after colorectal surgery were identified through searching multiple online databases until June 2023. Pairs of reviewers independently evaluated the quality using an adapted version of the Consensus on Health Economic Criteria list. Extracted costs were converted to 2022 euros (€) and also adjusted for purchasing power disparities among countries.

**Results:**

From 1980 unique abstracts, 59 full-text publications were assessed for eligibility, and 17 studies were included in the review. The incremental costs of AL after correcting for purchasing power disparity ranged from €2250 (+39.9%, Romania) to €83,633 (+ 513.1%, Brazil). Incremental costs were mainly driven by hospital (re)admission, intensive care stay, and reinterventions. Only one study estimated the economic societal burden of AL between €1.9 and €6.1 million.

**Conclusions:**

AL imposes a significant financial burden on hospitals and social care systems. The magnitude of costs varies greatly across countries and data on the societal burden and non-medical costs are scarce. Adherence to international reporting standards is essential to understand international disparities and to externally validate reported cost estimates.

**Supplementary Information:**

The online version contains supplementary material available at 10.1007/s10151-024-02932-4.

## Introduction

Anastomotic leakage remains a significant and burdensome complication following colorectal surgery. It is associated with a wide range of clinical consequences, including increased morbidity, need for reoperations, temporary or definitive ostomy creation, prolonged hospitalization, negative oncologic impact, and even mortality [1–3]. Moreover, the occurrence of AL can impose a substantial socioeconomic burden on both healthcare institutions and society at large resulting from the exhaustive resource utilization [4]. The reporting of financial costs attributable to AL in a health economic evaluation is scarce and costs may vary according to differences among international healthcare systems. Previous economic analysis of post-surgical complications following major procedures, including colorectal, hepatopancreatobiliary, and bariatric surgeries, indicated that the overall costs can surpass 100,000 euros per individual case [5]. The extent of the societal burden in terms of direct and indirect costs borne by patients and their family, costs due to productivity loss in work, and rehabilitation costs remains largely unknown but is certainly extensive.

As global healthcare expenditures continue to rise, there is an increasing need for accurate and reliable cost calculations to support efforts to keep healthcare affordable and provide data for cost-effectiveness analyses [6]. Decision-making regarding the implementation of novel preventive measures and different treatment strategies for AL is largely driven by this type of health economic evaluation. Many approaches toward reducing AL rates are emerging, including the use of intraoperative perfusion assessment with fluorescence angiography, or algorithms aimed at identifying modifiable perioperative risk factors [7, 8]. The clinical adoption of such strategies demand assessment of efficacy of the interventions and reliable cost estimates to accurately judge its cost-effectiveness [9]. Just as crucially, AL has a devastating impact on our patients and causes a clinically relevant decrease in health-related quality of life [10], which must be taken into account beyond mere financial considerations. However, numerous cost studies referenced in literature regarding AL following colorectal surgery are either outdated or tailored to particular countries or settings. There exists a critical necessity for an updated and comprehensive review encompassing all relevant studies assessing the economic implications associated with AL following colorectal surgery. The objective of this study was to systematically review the available literature on the (socio)economic costs of AL following colorectal surgery, appraise their quality, compare reported outcomes, and identify knowledge gaps by which future cost studies could be improved.

## Methods

This systematic literature review followed the reporting guidelines outlined in the PRISMA statement (2020) and was PROSPERO registered (registration ID: CRD42022367453) [11]. It should be noted that not all PRISMA reporting items were fully applicable for the purpose of this review.

### Search strategy

A literature search was performed in PubMed (MEDLINE), Embase (OVID), and the Cochrane Central Register of Controlled Trials. We identified studies in English or Germanic language originating from various global healthcare settings. The search captured studies dating from inception of the databases up to July 2023. The search strategy was assisted by a clinical librarian. MeSH terms and keywords related to colorectal surgery *and* anastomotic leakage *and* health economic evaluation were included in the search. The full reproducible search strategy is presented in supplementary file 1. 

### Eligibility

The objective was to gather the globally available literature that report the (socio)economic costs of AL following colorectal surgery.

The inclusion criterion for the review was an original article reporting an estimate of the (socio)economic costs resulting from AL following colorectal surgery.

Exclusion criteria were:

- Papers reporting sole costs of patients with AL without exploration of incremental costs of patients without AL or uncomplicated courses.
- Papers modeling costs on the basis of secondary data (existing data from previous studies, which are potentially included).
- Papers reporting effect on hospital net revenue rather than incremental costs due to AL.

### Study selection

**Fig. 1:**
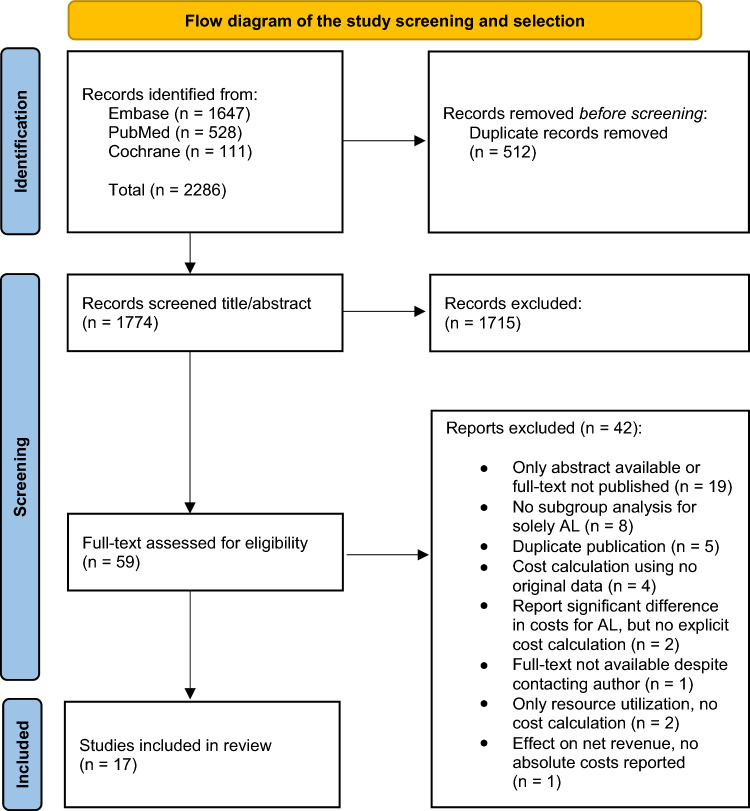
Flow diagram of the study screening and selection. *From:* Page et al. [11]. 10.1136/bmj.n71

Articles were screened independently by two authors (D.N. and K.W.) using the Rayyan online Tool for Systematic Literature Reviews [12] in two phases: title/abstract screening and full-text screening. After reaching consensus in each phase, eligible articles were gathered including reasons for exclusion. If consensus was not reached, decision-making was based on a third author’s expert advice (R.H., W.L., or M.J.P.). The final inclusion of articles and reasons for exclusion are presented in a flowchart (Fig. 1).

### Data collection process

Data were extracted by D.N. and K.W. independently from each article and comprised study design or type of health economic evaluation, study population and demographics, surgical procedure(s), AL severity classification, time horizon for the cost calculation, data source(s), included costs units or resources, resource utilization, primary objective and outcome measure, chosen perspective (e.g., societal, hospital, insurance company), societal costs, incidence of AL, and finally, our main outcome, the incremental costs due to AL. If both non-adjusted and adjusted outcomes (after adjusting for covariates by multivariate analysis or propensity score matching by some studies) were presented in articles, only the adjusted outcomes were presented in this review. Furthermore, if both top-down or bottom-up calculations were performed in the studies, only the bottom-up calculations were presented in this review, as these calculations are deemed more precise according to expert consensus [13].

To effectuate comparability, costs presented in each article were initially converted from prices of the reported reference year (or the year in which the article was accepted if no reference year was mentioned) to 2022 prices. This conversion was made by applying annual inflation rates specific to the country associated with each study (Inflation Tool—CPI Calculator & Inflation Rates) [14]. Furthermore, to assess the societal impact of costs, prices were corrected for the purchasing power disparities among different countries. This was done by recalibrating prices using purchasing power parities (PPPs) accessible on the Organisation for Economic Co-operation and Development (OECD) website (https://www.oecd.org) [15]. All costs were converted to euros (€) by utilizing the average 2022 exchange rates between the reported local currencies and the euro (€) as provided on the European Central Bank (ECB) website (https://www.ecb.europa.eu/) [16]. Costs were rounded to zero decimal places.

### Quality assessment

The methodological characteristics of the included studies were assessed using an adapted version of the Consensus on Health Economic Criteria (CHEC) criteria list, which was originally developed for methodological assessment of full health economic evaluations to aid systematic reviews [17]. As our study primarily focuses on the quality of the cost-analysis aspect within health economic evaluations, rather than pursuing a full health economic evaluation encompassing cost-effectiveness or cost-utility, 14 of the original 19 listed items were included in the final assessment of articles. This list is available in supplementary file #2. CHEC items aimed solely at full health economic evaluations (e.g., cost-effectiveness, cost-utility) were excluded from the original CHEC list. The assessment was done independently by D.N. and K.W. When in disagreement, other contributing authors were consulted for definitive assessment (R.H., W.L., M.J.P.). Each question is answered with either “yes” or “no” and points were assigned as follows: no, 0 points; yes, 1 point. Since the CHEC list does not specify summary scores, we established the scoring thresholds to assess the methodological quality of the studies. A total score ≥ 10.5 was deemed indicative of high quality, a score of 7–10 moderate-quality, and a score < 7 low-quality, as earlier demonstrated by Deviandri et al. [18], but extrapolated to a CHEC list with 14 items instead of 19 items.

### Definitions

The included costs analyses were categorized on the basis of the costing methodologies applied. Briefly, two main categories are used to classify the costing method:

*Top-down costing*: costs are estimated at a higher level by using aggregated data and averages, often involving tariffs. This method provides an overall view of costs without going into detailed cost components. For instance, systems such as diagnosis-related groups (DRG) can provide payment tariffs that can be used in top-down calculations by grouping specific patient populations [19]. Studies were classified as top-down if they made use of tariffs or if they did not report individual costs components.

*Bottom-up costing*: costs are estimated by adding up individual cost components, providing a more detailed breakdown of specific resources and services consumed. Applied to hospital costs, this often involves breaking down costs into specific expenses attributed to medical interventions, nursing care, medication, diagnostic tests, and other services. Studies were classified as bottom-up if they reported a breakdown of individual cost components aggregating to the final cost estimate.

Furthermore, a distinction is made between partial and full health economic evaluations, which was defined as:

*Full health economic evaluation:* specific study designs that compare costs and consequences (or effects), e.g., cost-effectiveness analysis, cost-utility analysis, cost-benefit analysis. [20]

*Partial health economic analysis*: one-sided analysis of costs attributable to a certain healthcare condition or event, without establishing the correlation between costs and consequences or effects, e.g., cost analysis, cost-of-illness analysis.

## Results

### Study selection

The search strategy retrieved 2286 articles, of which 1774 original articles remained after deduplication. The screening process is schematically presented in a flow diagram including the reasons for exclusion (Fig. 1). A total of 17 articles were found eligible for inclusion in the final review.

### General characteristics of included studies

The study specification and demographics of the included articles are presented in Table 1. The majority of the studies originated from the USA (*n* = 4), and the remaining were conducted in Germany (*n* = 3), Austria (*n* = 1), China (*n* = 1), the UK (*n* = 1), Italy (*n* = 1), Romania (*n* = 1), Switzerland (*n* = 1), South Korea (*n* = 1), Brazil (*n* = 1), Canada (*n* = 1), and Japan (*n* = 1). All studies were partial economic evaluations and two studies [21, 22] had an alternate primary objective other than quantifying the costs of AL. The level of detail in specifying the study population varies among studies; seven studies exclusively focused on lower (rectal) resections [ultra-low anterior resection (uLAR), low anterior resection (LAR), or anterior resection (AR)] [4, 23–28], while the remaining ten studies group all colorectal procedures together [21, 22, 29–36]. The time horizons of the studies ranged from 6 months to 13 years and all of the studies reported costs from the hospital perspective.

**Table 1:** Study characteristics

First author (year)	Country	Study population	Total included patients (*n*)	Level	Study perspective	Time horizon	Data source(s)	Study design
Ashraf (2013)	UK	AR for colorectal cancer	285	National	Hospital and societal	3 years	Internal hospital database	Retrospective cohort study
							Remuneration tariffs based on HRG; and (ii) DH reference index costing	
Ammann (2019)	USA	LAR for colorectal cancer	7479 and 2259 (Premier and Optum)	National	Hospital and insurance reimbursement	5 years	Premier Healthcare Database (700 US Hospitals) Health insurance claims data (Optum)	Retrospective cohort study
Bai (2022)	China	Left-sided colorectal resections with anastomosis	168	Regional and national	Hospital	6 months	Chinese regional database (China HIS Database)	Retrospective cohort study
Bogner (2022)	Germany	Elective colorectal resections (ileocecal resection, hemicolectomy, sigmoid resection, rectal resection, colonic segmental resection, colectomy, Hartmann’s reconstruction)	308	Regional	Hospital	3 years and 4 months	Internal hospital database Pricing data through German G-DRG system	Retrospective cohort study
Capolupo (2022)	Italy	Colorectal resections (colectomy, hemicolectomy, or rectum resection) with anastomosis	317	Regional	Hospital and insurance reimbursement	2 years	Internal hospital database	Retrospective cohort study
Elthes (2020)	Romania	Colorectal cancer resections (not further specified)	120	Regional	Hospital	2 years and 6 months	Internal hospital database	Retrospective cohort study
Fortin (2021)	USA	Colorectal resections (Hemicolectomy, LAR, sigmoidectomy)	13,167	National	Hospital	2 years and 3 months	Premier Healthcare Database (700 US hospitals)	Retrospective cohort study
First author (year)	Country	Study population	Total included patients (*n*)	Level	Study perspective	Time horizon	Data source(s)	Study design
Hammond (2014)	USA	Colorectal resections (colectomy, hemicolectomy, rectum resection) with anastomosis	106,053	National	Hospital	3 years	Premier Healthcare Database (600 US hospitals)	Retrospective cohort study
Kang (2022)	South Korea	AR, LAR, or uLAR for colorectal cancer using manual circular stapler	120,245	National	Hospital and insurance reimbursement	13 years	Health Insurance Review and Assessment Service (HIRA) national database National health claims reimbursement data	Retrospective cohort study
Kumamaru (2022)	Japan	LAR for rectal cancer	15,187	National	Hospital	2 years	National Clinical Database (NCD) Japan	Retrospective cohort study
Koperna (2003)	Austria	LAR for rectal cancer	70	Regional	Hospital	3 years	Internal hospital database	Retrospective cohort study
la Regina (2019)	Switzerland	Colorectal resections with anastomosis	95	Regional	Hospital	2 years	Internal hospital database	Retrospective cohort study
Lee (2019)	USA	Bariatric and colorectal resections	239,350 and 19,958 Medicare and commercial	National	Hospital and commercial and public insurance reimbursement	2 years and 8 months	Medicare Fee-for-Service claims data OptumInsight Inc. commercial claims data	Retrospective cohort study
Meyer (2002)	Germany	Curative resections for rectal cancer	11	Regional	Hospital	5 years	Single center, single surgeon, 5-year follow-up	Retrospective cohort study
Ribeiro (2019)	Brazil	LAR	337	National	Hospital	2 years	National private healthcare administrative database	Retrospective cohort study
Springer (2019)	Canada	Colorectal resections with anastomosis	108,304	National	Hospital	7 years	National discharge abstract database, excluding Quebec	Retrospective cohort study
Weber (2023)	Germany	Colon resections and sphincter-preserving rectal resections	690,690	National	Hospital	6 years	Federal Statistical Office (DESTATIS)	Retrospective cohort study

*LAR* low anterior resection, *AR* anterior resection, *uLAR* ultra-low anterior resection

Additionally, taking a broader perspective, three studies also reported insurance reimbursement costs [23, 29, 34], one study reported patient out-of-pocket costs [24], and one study reported estimated societal burden.

### Methodological quality

**Fig. 2:**
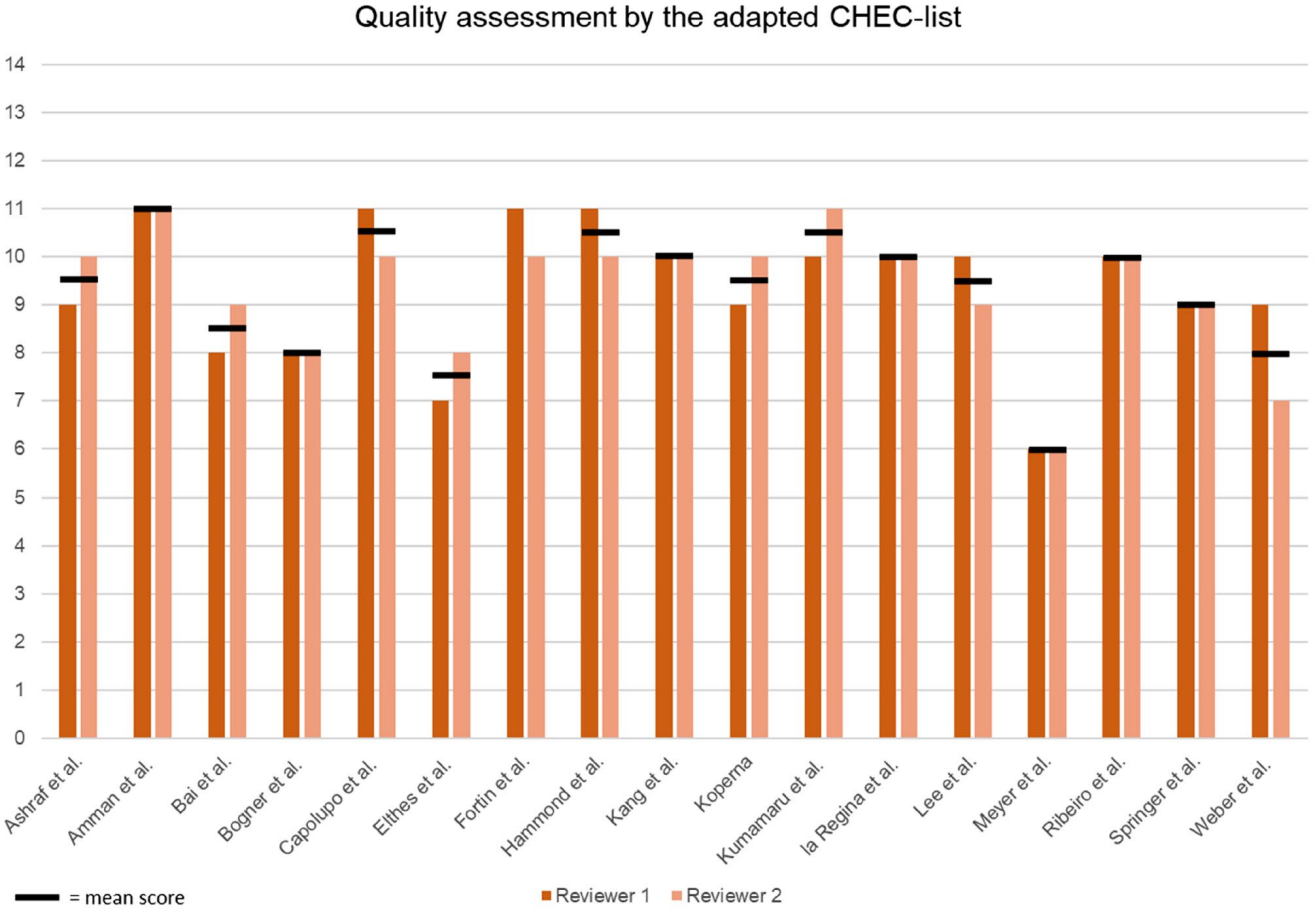
Quality assessment of the included studies by the adapted CHEC list. The mean score of reviewer 1 and 2 is depicted by the horizontal black line. An overview of the included scoring items from the original CHEC list can be found in supplementary file 2

Studies were scored for methodological quality using the adapted version of the CHEC list (Supplementary file 2). The scores of the included studies are displayed in Fig. 2 and ranged from 6 to 11. In total, 5 studies were classified as high quality, 11 as moderate quality, and 1 as low quality.

### Population characteristics and clinical outcome

Table 2 describes the population characteristics and clinical outcomes of the included studies. Anastomotic leakage rates ranged from 4.3% [26] to 29.8% [21]. Three studies reported the severity of AL either according to the International Study Group of Rectal Cancer classification Grade A–C [28, 30] or to the severity of peritoneal contamination [4], and 7 out of 17 studies reported the rate of diverting ostomy during index surgery [4, 22, 24, 26, 28, 29, 33], of which the highest reported rate was by Ashraf et al. [4] at 37.2% and the lowest rate by la Regina et al. [33] at 11.6%. The largest increase in index hospitalization length was seen in the report by Meyer [27], who reported a mean increase from 12.4 days to 63.3 days in cases in which AL occurred. Overall, intensive care unit (ICU) rates ranged from 5.3% to 52.5%, and this greatly increased for patients with AL across all studies that reported these rates. Readmission and mortality rates were increased in patients with AL in all studies that reported these figures. These rates after AL ranged from 3.7% to 29.0% (30-day readmission) [32, 34] and from 18.5% to 44.0% (90-day readmission) [21, 31]. The mortality rate ranged from 3.2% (30 days) to 25% (index admission) [4, 30] in all cases with AL and from 0% (index admission) to 11% (index and readmission) [32, 33] in patients without AL.

**Table 2:** Clinical outcome parameters reported in included studies

First author (year)	Country	Surgical approach (% if mentioned)	Anastomotic leakage *n* (%)	Stoma rate after surgery *n* (%)			Length of stay index hospitalization (days), mean (SD)	
				No AL	AL	Overall	No AL	AL
Ashraf (2013)	UK	Open (43.2) + laparoscopic (56.8)	31 (10.9%)	89 (35.2)	17 (53.1)	106 (37.2)	9.2 (6.9)	30.3 (21.6)
Ammann (2019)	USA	Open (76.1) + laparoscopic (6.0) + robotic (9.0) + laparoscopic conversion (8.8)	Premier cohort: 776 (10.4%) Optum cohort: 253 (11.2%)	NR	NR	NR	Premier 6.8 Optum 6.0	Premier 12.1 Optum 11.6
Bai (2022)	China	Open (11.3) + laparoscopic + robotic	50 (29.8%)	NR	NR	NR	14.1 (0.5)	23.9 (1.8)
Bogner (2022)	Germany	Open (42.5) + laparoscopic (23.7) + robotic (25.6) + laparoscopic conversion (6.2) + robotic conversion (1.9)	33(10.7%)	69 (25.1)	6 (18.2)	75 (24.4)	NR	NR
Capolupo (2022)	Italy	Open (27.4) + laparoscopic (72.6)	39 (12.3%)	58 (20.9)	15 (38.5)	73 (23.0)	10.3 (5.6)	20.1 (10.7)
Elthes (2020)	Romania	Open	12 (10%)	NR	NR	NR	10.5	17.4
Fortin (2021)	USA	Open (26.4) + laparoscopic (48.1) + robotic (25.5)	690 (5.24%)	NR	NR	NR	4.99	7.79
Hammond (2014)	USA	Open + laparoscopic	6174 (6.2%)	NR	NR	NR	15.7 (16.7)	23.0 (23.3)
Kang (2022)	South Korea	Open (25.9) + laparoscopic (74.1)	7194 (6.0%)	17.894 (15.8)	1.953 (27.2)	19.847 (16.5)	14.2 (8.4)	16.8 (13.2)

First author (year)	Country	ICU admission during index hospitalization *n* (%)			Readmission, *n* (%)		Mortality *n* (%)		
		No AL	AL	Overall	No AL	AL	No AL	AL	Overall
Ashraf (2013)	UK	9 (3.6)	6 (19.4)	15 (5.3)	NR	NR	30-day: 5 (2.0)	30-day: 1 (3.2)	30-day: 6 (2.1)
Ammann (2019)	USA	NR	NR	NR	Premier 90-day: 19.5% Optum 90-day: 26.2%	Premier 90-day: 26.9% Optum 90-day: 33.1%	NR	NR	NR
Bai (2022)	China	NR	NR	NR	90-day: 18.6%	90-day: 44.0%	NR	NR	NR
Bogner (2022)	Germany	NR	NR	NR	NR	NR	NR	NR	30-day: 1 (0.3)
Capolupo (2022)	Italy	25 (9.0)	15 (38.5)	40 (12.6)	NR	NR	NR	NR	NR
Elthes (2020)	Romania	19 (17.6)	10 (83.3)	29 (24.2)	30-day: 11 (10.2)	30-day: 3 (25)	Index: 4 (3.7)	Index: 3 (25)	Index: 7 (5.8)
Fortin (2021)	USA	NR	NR	NR	30-day: 8.7% 60-day: 11.6% 90-day: 13.4%	30-day: 12.2% 60-day: 16.0% 90-day: 18.5%	NR	NR	NR
Hammond (2014)	USA	NR	NR	NR	30-day: 9278 (9.9)	30-day: 1801 (29.2)	Index + readmission 695 (11)	Index + readmission 714 (12)	Index + readmission 1409 (11)
Kang (2022)	South Korea	NR	NR	NR	NR	NR	NR	NR	NR

First author (year)	Country	Surgical approach	Anastomotic leakage *n* (%)	Stoma rate after surgery *n* (%)			Length of stay index hospitalization (days), mean (SD)	
				No AL	AL	Overall	No AL	AL
Kumamaru (2022)	Japan	Open (28.7) + laparoscopic (71.3)	1405 (9.3%)	NR	NR	NR	Median (IQR): 13 (10–17)	Median (IQR): 37 (27–51)
Koperna (2003)	Austria	Not stated	3 (4.3%)	NR	NR	19 (27.1)	NR	45.3 (8.3)
la Regina (2019)	Switzerland	Open (20.0) + laparoscopic (80.0)	8 (8.4%)	10 (11.5)	1 (12.5)	11 (11.6)	9.7 (3.0)	29.1 (9.9)
Lee (2019)	USA	Open + laparoscopic	Medicare 11.966 (5.0%) Commercial 1012 (5.1%)	NR	NR	NR	Medicare: 7 (SD NR) Commercial: 5 (SD NR)	Medicare: 19 (SD NR) Commercial: 7 (SD NR)
Meyer (2002)	Germany	Not stated	3 (27.3%)	NR	NR	NR	12.4 (2.4)	63.3 (21.4)
Ribeiro (2019)	Brazil	Open (63.5) + laparoscopic (36.5)	23 (6.8%)	45 (14.3)	11 (47.8)	56 (16.6)	7.5 (6.7)	39.6 (48.6)
Springer (2019)	Canada	Open (68.0) + laparoscopic (32.0)	5847 (5.4%)	NR	NR	NR	NR	NR
Weber (2023)	Germany	Not stated	47,453 (6.87%)	NR	NR	NR	NR	NR

First author (year)	Country	ICU admission *n* (%)			Readmission (%)		Mortality *n* (%)		
		No AL	AL	Overall	No AL	AL	No AL	AL	Overall
Kumamaru (2022)	Japan	NR	NR	NR	NR	NR	NR	NR	NR
Koperna (2003)	Austria	NR	Duration: mean (SD): 16.7 (5.2)	NR	NR	NR	NR	NR	NR
la Regina (2019)	Switzerland	NR	NR	NR	NR	NR	Index: 0	Index: 1 (12.5)	Index: 1 (1.1)
Lee (2019)	USA	NR	NR	NR	Medicare: 30-day: 3647 (1.52) All: 33.769 (14.1) Commercial: 30-day: 294 (1.5) All: 1689 (8.5)	Medicare: 30-day: 317 (3.7) All: 1849 (29.6) Commercial: 30-day: 41 (5.7) All: 93 (12.9)	NR	NR	NR
Meyer (2002)	Germany	0	3 (100)	3 (27.3)	NR	NR	NR	NR	NR
Ribeiro (2019)	Brazil	158 (50.3)	19 (82.6)	177 (52.5)	30-day: 26 (8.3)	30-day: 6 (26.1)	Index: 4 (1.3)	Index: 5 (21.7)	Index: 9 (2.7)
Springer (2019)	Canada	NR	NR	Duration: Mean (SD) 1.2 (4.9)	NR	NR	NR	NR	NR
Weber (2023)	Germany	NR	NR	NR	NR	NR	Colon (2018): index 7.1% Rectal (2018): index 3.5%	Colon (2018): index 20.1% Rectal: index 11.3% (2018)	NR

Data are presented as stated in the top row unless specified differently in the relevant row

*NR* not reported, *ICU* intensive care unit, *AL* anastomotic leakage

### Economic outcomes

Table 3 describes the economic outcomes reported in the various studies. The time horizon over which the costs of AL were calculated ranged from only index admission costs to index admission + 30 days, 1 year, or 5 years. The highest incremental costs for AL, after correcting for inflation and purchasing power disparities, were reported by Ribeiro et al. [28] (Brazil) for the index admission costs + 30 days at 83,633€, marking the highest increase percentage-wise at 513.1% as well. Conversely, Elthes et al. [30] (Romania) calculated the lowest incremental costs looking only at index admission costs at 2250€ or a 39.9% increase. Percentage-wise the lowest increase in costs of AL was demonstrated by Bai et al. [21] with a 29.1% increase. In eight studies, the costs doubled or more than doubled (up to a fivefold increase) when AL occurred [4, 26–29, 33, 36].

**Table 3:** Economic outcomes

First author (year)	Country	Time horizon of included costs	Costing method	Anastomotic leakage, *n* (%)	Control group, *n*	Total costs without AL, per patient	Incremental costs AL, per patient	PPP-corrected incremental costs AL, per patient	Main cost drivers	Conclusion
*Low resections for colorectal cancer (AR, uLAR, or LAR)*										
Ashraf (2013)	UK	Index admission costs	Bottom-up (DH-index) + top-down	31 (10.9%)	254	Hospital costs: 9302€	16,046€ (+172.5%)	19,081€	Not specified	The estimated economic burden of anastomotic leakage following AR is approximately double that of the remunerated tariff
Ammann (2019)	USA	Index admission costs + 90 days post-discharge costs	Top-down	Premier cohort: 776 (10.4%) Optum cohort: 253 (11.2%)	Premier cohort: 6703 Optum cohort: 2006	Hospital billing: 35,265€ (index) 65,059€ (index + 90d) Hospital costs: 24,725€ (index)	20,217€ (+57.3%) 24,397€ (+37.5%) 13,401€ (+54.2%)	24,397 20,217€ 13,401€	Not specified	In-hospital infection, anastomotic leak, and bleeding were associated with a substantial economic burden, for both hospitals and payers, in patients undergoing LAR for colorectal cancer
Kang (2022)	South Korea	Index admission + 30-day readmissions	Top-down	7194 (6.0%)	113,051	Hospital billing: 8829€	5273€ (+59.7%)	7289€	Not specified	AL is associated with a significantly increased economic burden in the nationwide dataset
Kumamaru (2022)	Japan	Index admission	Bottom-up	1405 (9.3%)	10,879	Hospital costs: 16,618€	10,662€ (+ 64.2%)	10,927€	Not specified	Postoperative complications and their severity are strongly associated with increases in hospital costs, the utilization of health-care resources, and poLOS
Koperna (2003)	Austria	Index admission + 1-year costs	Bottom-up	3 (4.3%)	67	Hospital costs: 16,133€	52,157€ (+323.3%)	67,944€	ICU stay Readmission Reoperation	AL is the most important cost driver
Meyer (2002)	Germany	Index + 5-years costs	Bottom-up	3 (27.3%)	8	Hospital costs: 24,518€	34,766€ (+141.8%)	45,352€	Not specified	A complicated course is associated with a considerable increase in costs
Ribeiro (2019)	Brazil	Index costs + 30-day readmissions	Top-down	23 (6.8%)	314	Hospital billing: 7982€	40,958€ (+513.1%)	83,633€	Not specified	AL leads to worse clinical outcomes and increases hospital costs by 4.66 times
*Low + high resections for colorectal cancer (LAR, ileocecal resection, hemicolectomy, sigmoid resection, colectomy)*										
Elthes (2020)	Romania	Index admission costs	Bottom-up + top-down (DRGs)	12 (10%)	108	Hospital costs: 2132€	851€ (+39.9%)	2250€	Not specified	An important economic burden can also be noticed, increasing hospitalization costs by 1.66 times and resulting in significant financial loss for the hospital
*Low + high colorectal resections for benign + malignant disease (LAR, ileocecal resection, hemicolectomy, sigmoid resection, colectomy)*										
Bai (2022)	China	Index admission costs	Top-down	50 (29.8%)	118	Hospital billing: 8725€	2537€ (+29.1%)	4241€	Not specified	ECP is favored over manual circular staplers in terms of both the clinical and economic benefits for left-sided colorectal anastomoses in hospital settings in China
Bogner (2022)	Germany	Index admission costs + readmissions (no time horizon stated)	Top-down	33 (10.7%)	Not specified	Hospital billing: 12,669€	7728€ (+ 61.0%)	10,081€	Not specified	SDD significantly reduces the incidence of AL and SSI and saves costs for the general healthcare system
Capolupo (2022)	Italy	Index admission costs	Bottom-up	39 (12.3%)	278	Hospital costs: 7672€	8247€ (+107.5%)	12,531€	ICU stay Pharmaceuticals Ward stay Hospital consultations Operating room	In our Italian center, the incidence of AL following colorectal surgery increased total inpatient cost by 108% and raised the odds of reoperation and ICU stay by 25 and 8 times, respectively, compared with patients without AL
Fortin (2021)	USA	Index admission + 90-day readmissions costs	Top-down	690 (5.24%)	12.477	Hospital costs: 22,517€	17,410€ (+77.3%)	17,410€	Not specified	In this analysis of patients undergoing left-sided colorectal reconstructions involving a manual circular stapler, circular anastomotic complications were associated with adverse economic consequences
Hammond (2014)	USA	Index admission + 30-day readmissions	Top-down	6174 (6.2%)	6174 (after PSM)	Hospital costs: 52,018€	33,573€ (+64.5%)	33,573€	All included units	AL following colorectal surgery increase the total clinical and economic burden by a factor of 0.6–1.9
la Regina (2019)	Switzerland	Index admission costs	Bottom-up	8 (8.4%)	87	Hospital costs: 18,185€	55,991€ (+307.9%)	58,258€	ICU stay Operating room Physician salaries Nursing care	AL has a large negative influence on medical resource utilization. The complication-related increase of DRG reimbursement is not sufficient to cover increased costs, so that every complicated case represents a financial burden for the hospital
Lee (2019)	USA	Index + 30-days costs	Top-down	Medicare 11,966 (5.0%) Commercial 1012 (5.1%)	227,384 18,973	Hospital billing: Commercial: 40,269€ Medicare: 28,920€	35,920€ (+89.2%) 42,859€ (+148.2%)	35,920€ 42,859€	Not specified	AL are associated with increased costs and LOS during the initial procedure. They are also associated with a significantly greater readmission rate, LOS, and overall costs when comparing leak versus no leak readmissions 30 days after the initial stay
Springer (2019)	Canada	Index admission costs	Top-down	5847 (5.4%)	Baseline definition: 50-year–old man who underwent an open colon resection for benign disease with no comorbidities or complications	Hospital billing: 8099€	7953€ (+9.5%)	8444€	Not specified	Medical and surgical complications (especially those requiring reoperation) are major drivers of increased resource use
Weber (2023)	Germany	Index admissions costs	Top-down	47,453 (6.87%)	643,237	Hospital billing: 16,574€	22,989€ (+138.7%)	29,988€	Not specified	A significant increase in the hospital reimbursement sum for cases with AL compared with cases without AL can be seen

Prices are presented in 2022 EUR (€) after correction for inflation and conversion to euro when presented in other currency. Prices are corrected for purchasing power per country using purchasing power parities (PPP) when stated as such

*Hospital billing* refers to the charges billed by the hospital for specific services provided, including medical procedures, medications, and other associated expenses

*Hospital costs* represent the true expenses incurred by the hospital for delivering the required healthcare services, taking into account factors such as personnel salaries, medical supplies, facility maintenance, and overhead expenses

Seven studies estimated the incremental costs of anastomotic leakage specifically in a homogeneous group after undergoing a lower resection for colorectal cancer (AR, uLAR, or LAR). These costs ranged from 7289€ (index admission + 30 days) [25] to 83,633€ (index admission + 30 days) [28]. One study encompassed both higher and lower resections for colorectal cancer in their cost estimation of anastomotic leakage, averaging 2250€ solely for the index admission [30]. The remaining nine studies estimated the costs in an aggregated group consisting of colon and rectal resections for both benign and malignant diseases. These costs ranged from 4241€ (index admission costs) to 58,258€ (index admission costs). The study by Ashraf et al. [4] conducted in the United Kingdom was the only study to report an estimated societal burden valued at €1.9 million to €6.1 million (€ 2022 corrected for inflation and purchasing power).

**Fig. 3:**
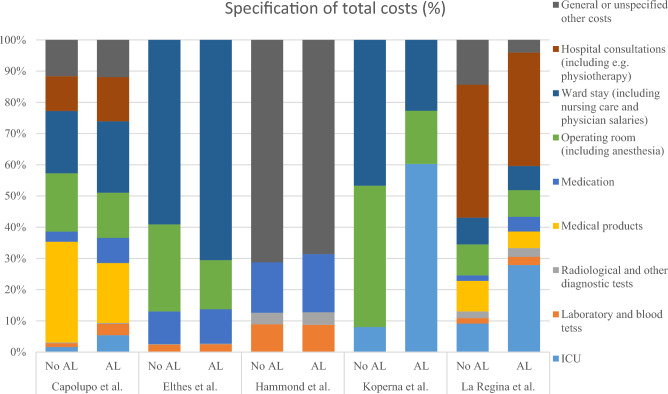
Specification of costs of AL compared with no AL. *AL* anastomotic leakage, *ICU* intensive care unit, *NR* not reported. General or unspecified costs were mentioned in the referred articles as: “costs other departments,” “building cost management,” “external service,” “administrative costs.” All costs are presented in 2022 euros (€) with proportions (%) of total costs horizontally presented. Costs are not corrected for purchasing power disparities

Figure 3 shows a specification of the total hospitals costs of all the studies that reported a breakdown of the costs. Only three studies included ICU costs in their specifications, but in all of them, ICU represented a more substantial portion of the costs when AL occurred. Moreover, in some studies, ward stay and medication appeared to account for a larger proportion of the total costs when AL occurred, although this pattern did not hold true for all studies.

## Discussion

This is the first systematic review to provide a global perspective on the costs associated with AL, one of the most feared complications following colorectal surgery. The current literature on cost analyses of AL was found to be highly heterogeneous in both methodology and the reporting of outcomes. However, regardless of international healthcare setting, AL imposes a significant financial burden on hospitals, healthcare insurance, and society at large. Important cost drivers were hospital (re)admission, intensive care stay, and reinterventions. Literature on the estimated societal burden and direct non-medical costs such as extramural expenses borne by the patient, family, and other sectors are scarce or unreported.

The magnitude of AL-related expenses endured by the hospital varied greatly across countries and individual studies. Elthes et al. [30] described that the incremental costs for AL in a Romanian cohort of colorectal cancer resections were only 851€ (2250€ after correction for purchasing power disparities with PPP). This figure is an outlier and remarkably lower than the estimation by Ribeiro et al. [28] in Brazil, who estimated the incremental costs of AL after low anterior resection at 40,958€ or 83,633€ after correcting with PPP—a factor 37.2 higher than the Romanian cohort. This variance highlights significant differences in expenses among countries and healthcare settings, but also suggests diverse approaches in estimating incremental costs. Firstly, the time horizon for the cost calculation is an important factor that influences the magnitude of the reported incremental costs. Secondly, the difference in study populations can be another factor contributing to the significant variation in AL related costs between the studies. Specifically, seven of the included studies focused on a distinct cohort of patients with AL after low resection for colorectal cancer. One study encompassed both low and high resections for colorectal cancer, while nine studies encompassed a broader patient population experiencing AL following both benign and malignant colorectal surgery. It is noteworthy that costs associated with AL may vary between rectal and colon cancer surgeries. For instance, AL after rectal cancer surgery has a lower 30-day mortality rate compared with AL after colon cancer surgery (5.7% versus 16.4%), likely resulting in reduced ICU-related costs [37, 38]. Thirdly, the individual cost units included in the calculation determine the resulting sum, which also differs across studies. Several of the included studies adopted a (partially) bottom-up approach for cost estimation, elaborating on individual cost units, such as healthcare resources, procedures, or interventions, which were then aggregated to calculate the total costs [25–27, 29, 30, 33]. Conversely, the most frequent costing method was top-down, estimating costs at a higher level often using national or regional average tariffs [21–24, 28, 31, 32, 34, 35]. In this top-down approach, the specific individual cost units contributing to the final estimate often remain unclear, which limits the comparability of the studies. This limitation does not necessarily constrain the overall quality of these studies, as all included studies were partial economic evaluations and some pursued primary objectives other than cost estimation for AL. For example, Bai et al. [21] aimed to examine the clinical benefits of a new powered circular stapler, using a cost-analysis of AL as an argument for economic benefit.

A limitation of the majority of the included studies was the limited time span. The cost calculation was based on AL identified either during index hospitalization [4, 21, 29, 30, 33, 35, 36] or within 30 days postoperative [22, 24, 28, 32, 34] in the majority of the studies. However, restorative surgical procedures after AL are usually performed more than 30 days after index surgery, so these costs were not included in most studies. Prior literature also shows that a substantial amount of leaks are diagnosed 1–3 months to even 1 year after rectal cancer resections, indicating that a 30-day time span is insufficient to measure the financial impact of AL and ideally should cover the whole first year after the index surgery [39]. Stoma-related costs are another factor contributing to an underestimation of actual costs. A recent Swedish study revealed that annual stoma-related costs, including both direct and indirect costs, approximate 35,400€ and 37,400€ for a colostomy and ileostomy, respectively [40]. A previous report indicates that over half of patients who experience late-detected AL may develop a chronic presacral sinus, requiring an ostomy and major restorative surgery [39]. The true financial burden of AL seems to exceed the estimates based on the current literature, which should be emphasized when interpreting these data.

In line with expectations, the studies identified costly hospital (re)admissions, intensive care admission, and reinterventions as major cost drivers. Interestingly, these studies reported contrasting results regarding increase of hospitalization due to AL. Between included studies the reported increase of hospitalization length for patients with AL varied from threefold to fivefold [4, 25, 33, 34], indicating clinical variances in treatment of AL as well as possible difference in severity of AL between studies. This limits generalizability of the current results. However, it underlines that treatment strategies for AL that reduce hospitalization and reinterventions are likely to be cost-effective.

Novel strategies to prevent AL, such as intraoperative perfusion assessment with fluorescence angiography, or strategies to diagnose and treat AL early and effectively, are emerging and they necessitate comprehensive cost-effectiveness assessment for their clinical implementation. This systematic review can serve as a valuable resource for conducting cost-effectiveness studies by offering an overview of the potential types and magnitudes of costs that can be reduced in various countries worldwide. Nevertheless, researchers need to bear in mind that certain expenses, particularly those associated with long-term AL, are frequently missing in the reported studies and these costs will contribute substantially to overall AL-related costs. Longstanding unhealed anastomotic defects are known to result in chronic pelvic sepsis and poor bowel function with major low anterior resection syndrome, and will probably require major reinterventions in the long term. Furthermore, unhealed AL will result in a higher rate of end colostomies and impact social care systems, ultimately leading to a substantial rise in costs [40]. It is crucial to acknowledge that treatment approaches aimed at reducing long-term AL, which may initially result in heightened short-term costs, ultimately yield enhanced outcomes, thereby leading to substantial cost reductions in the long term.

The inclusion of costs from a societal perspective in studies investigating the expenses related to AL is scarce. Ashraf et al. [4] calculated the costs of AL based on internal hospital data, but subsequently extrapolated their costs to a national financial burden by using publicly available epidemiology figures. While it remains uncertain whether their data are transferable to other UK (NHS) hospitals, the reported estimates align with the plea for conducting health economic analyses from a societal perspective in the guideline [41, 42]. The only other study that reported social costs was by Kang et al. [24], who included out-of-pocket expenditures for patients not covered by insurance. Another shortcoming observed in all reported studies is the absence of certain indirect medical costs. Indirect medical costs originating from factors such as productivity loss, quality of life adjusted costs, extramural care, and private (non-hospital) expenses made by the patient or family are likely to make a significant contribution to the overall costs associated with AL. Furthermore, the studies did not factor in quality adjusted life years (QALYs) in their analyses, even though this forms an important driver for the implementation and adoption of novel, cost-effective treatment strategies. As a result, the complete societal burden of AL (including direct and indirect medical costs) remains to a great extent unknown and would be a valuable focus for future studies.

The methodological quality of the included studies, as assessed by our adapted CHEC list, was limited. Even after excluding certain items from the list that are applicable only in the context of a full health economic evaluation, 11 studies received moderate-quality scores, with one study even receiving a low-quality score. Several reports have evaluated the methodological quality of health economic analyses in different healthcare settings and have found that the quality was variable and overall suboptimal [43–45]. This is also reflected in our results. However, performing a formal quality assessment of the studies was complex due to the absence of complete health economic evaluations in the review. Therefore, we developed an adapted CHEC list to assess reporting items important for health economic evaluations across the studies, while acknowledging that it does not serve as a valid measure for methodological quality [17].

While this represents the first study summarizing the costs of AL after colorectal surgery, there are certain limitations to discuss. The main limitation is that we chose to include partial economic evaluations and studies with other primary objectives than estimating costs. Therefore, the selected studies cannot be held accountable for lacking methodological quality within health economics. Furthermore, few of the reported costs in the review can be disaggregated into individual cost units or linked to resource utilization, as is recommended for full health economic evaluations [13, 41]. Related to this, a formal quality and risk of bias assessment of the included studies was challenging, as there was a heterogeneity of study designs included in the review. Thus, an attempt was made to score individual studies on the basis of valued items for reporting in health economic evaluations, rather than methodological quality. However, this may hinder the assessment of the reliability of the presented costs for the reader.

The lack of comparability across cost analyses hinders a critical appraisal of the external validity of reported cost estimates in general. Nonetheless, the identification of this methodological heterogeneity stands as a pivotal outcome of our systematic review. In light of these findings, we strongly advocate that all economic evaluations should, to a certain extent, be standardized and adhere to international reporting standards [13]. We propose that authors should report at least all individual cost components, including direct and indirect costs. Additionally, an appropriate time horizon that includes all related costs should be considered. We would suggest a time horizon of at least 1 year in the case of investigating costs associated with AL after colorectal surgery. Complying with such principles not only improves understanding of international differences, but also ensures the accurate interpretation of a reported absolute cost estimate and its external validity.

### Conclusions

Anastomotic leakage after colorectal surgery imposes a substantial financial burden and drain on hospital resources. The magnitude of this burden varies greatly across regions and healthcare settings. The societal burden of these patients remains underreported in the current literature. However, together with center-specific cost estimates, this contributes to a reliable assessment of the cost-effectiveness of novel prevention and treatment strategies. Adherence to international reporting standards is essential to fully understand international disparities and to judge the external validity of reported cost estimates.

## Supplementary Information

Below is the link to the electronic supplementary material.

Supplementary file1 (DOCX 17 KB)

Supplementary file2 (DOCX 17 KB)

## Data Availability

The datasets generated and/or analyzed, analytic methods, and study materials that support the findings of this study are available from the corresponding author upon reasonable request.

## Declarations

### Preregistration

This study was preregistered in the independent, institutional registry PROSPERO (registration ID: CRD42022367453).
