# Supplementary material for: The economic impact of anastomotic leakage after colorectal surgery: a systematic review
Source: Tech Coloproctol. 2024 May 20;28(1):55. doi: 10.1007/s10151-024-02932-4 (PMC11106156; doi:10.1007/s10151-024-02932-4)
Supplement: Supplementary file 1 — Supplementary file1 (DOCX 17 KB) [file 10151_2024_2932_MOESM1_ESM.docx]

**Supplementary file 1. Search strategy**

**Initial search 26-9-2022:**

| Databases: |  |  |
| --- | --- | --- |
| PubMed, Embase (Ovid),  Cochrane CENTRAL Register of Controlled Trials | Before deduplication | After deduplication |
| Total | 2192 | 1722 |

Pubmed

496 hits:

("Colonic Diseases"[Mesh] OR "Rectal Diseases"[Mesh] OR "Colorectal Surgery"[Mesh] OR "Colon/surgery"[Mesh] OR "Rectum/surgery"[Mesh] OR "Colorectal Neoplasms"[Mesh] OR rectal*[tiab] OR rectum[tiab] OR colo*[tiab])

AND

("Anastomotic Leak"[Mesh] OR "Anastomosis, Surgical"[Mesh] OR anastomotic leak*[tiab] OR anastomosis leak*[tiab])

AND

("Economics"[Mesh] OR cost*[tiab] OR economic*[tiab] OR financ*[tiab])

**EMBASE (OVID):**

Database(s): **Embase Classic+Embase**1947 to 2022 September 23
Search Strategy:

| **#** | **Searches** | **Results** |
| --- | --- | --- |
| 1 | exp colorectal surgery/ | 27253 |
| 2 | exp colon disease/ | 667649 |
| 3 | exp rectum disease/ | 371089 |
| 4 | exp colorectal surgery/ | 27253 |
| 5 | exp colon/su [Surgery] | 3343 |
| 6 | exp rectum/su [Surgery] | 2701 |
| 7 | (rectal* or rectum or colo*).ti,ab,kf. | 1502442 |
| 8 | 1 or 2 or 3 or 4 or 5 or 6 or 7 | 1811645 |
| 9 | anastomosis leakage/ | 24210 |
| 10 | exp anastomosis/ | 283837 |
| 11 | (anastomo* adj3 leak*).ti,ab,kf. | 20361 |
| 12 | 9 or 10 or 11 | 303733 |
| 13 | exp economics/ | 250659 |
| 14 | exp "cost"/ | 391482 |
| 15 | exp economic evaluation/ | 339637 |
| 16 | (cost* or economic* or financ*).ti,ab,kf. | 1432058 |
| 17 | 13 or 14 or 15 or 16 | 1820354 |
| 18 | 8 and 12 and 17 | 1595 |

[Cochrane Central Register of Controlled Trials](https://www.cochranelibrary.com/)

Issue 8 of 12, August 2022

ID Search Hits

#1 (rectal* or rectum or colo*):ti,ab,kw 81798

#2 (anastomo* near/3 leak*):ti,ab,kw 1825

#3 (cost* or economic* or financ*):ti,ab,kw 99031

#4 #1 and #2 and #3 in Trials 101

**Search update 3-7-2023**

| Databases: |  |  |
| --- | --- | --- |
| PubMed, Embase (Ovid),  Cochrane CENTRAL Register of Controlled Trials | Before deduplication | After deduplication |
| Total | 94 | 52 |

Pubmed

32 hits:

("Colonic Diseases"[Mesh] OR "Rectal Diseases"[Mesh] OR "Colorectal Surgery"[Mesh] OR "Colon/surgery"[Mesh] OR "Rectum/surgery"[Mesh] OR "Colorectal Neoplasms"[Mesh] OR rectal*[tiab] OR rectum[tiab] OR colo*[tiab])

AND

("Anastomotic Leak"[Mesh] OR "Anastomosis, Surgical"[Mesh] OR anastomotic leak*[tiab] OR anastomosis leak*[tiab])

AND

("Economics"[Mesh] OR cost*[tiab] OR economic*[tiab] OR financ*[tiab])

AND

(2022/09/23:2023/07/03[Date - Completion])

**EMBASE (OVID):**

Database(s): **Embase Classic+Embase**2022 to 2023 July 3
Search Strategy:

| **#** | **Searches** | **Results** |
| --- | --- | --- |
| 1 | exp colorectal surgery/ | 29504 |
| 2 | exp colon disease/ | 783563 |
| 3 | exp rectum disease/ | 533966 |
| 4 | exp colorectal surgery/ | 29504 |
| 5 | exp colon/su [Surgery] | 3344 |
| 6 | exp rectum/su [Surgery] | 2701 |
| 7 | (rectal* or rectum or colo*).ti,ab,kf. | 1595882 |
| 8 | 1 or 2 or 3 or 4 or 5 or 6 or 7 | 1931250 |
| 9 | anastomosis leakage/ | 26414 |
| 10 | exp anastomosis/ | 302847 |
| 11 | (anastomo* adj3 leak*).ti,ab,kf. | 22036 |
| 12 | 9 or 10 or 11 | 324360 |
| 13 | exp economics/ | 252024 |
| 14 | exp "cost"/ | 408133 |
| 15 | exp economic evaluation/ | 355607 |
| 16 | (cost* or economic* or financ*).ti,ab,kf. | 1548851 |
| 17 | 13 or 14 or 15 or 16 | 1944199 |
| 18 | 8 and 12 and 17 | 1750 |
| 19 | limit 18 to dd=20220923-20230703 | 52 |

[Cochrane Central Register of Controlled Trials](https://www.cochranelibrary.com/)

Issue 7 of 12, July 2023

ID Search Hits

#1 (rectal* or rectum or colo*):ti,ab,kw 81798

#2 (anastomo* near/3 leak*):ti,ab,kw 1825

#3 (cost* or economic* or financ*):ti,ab,kw 99031

#4 #1 and #2 and #3 in Trials with Cochrane Library publication date Between Sep 2022 and Jul 2023, in Trials 10
