# Supplementary material for: The economic impact of anastomotic leakage after colorectal surgery: a systematic review
Source: Tech Coloproctol. 2024 May 20;28(1):55. doi: 10.1007/s10151-024-02932-4 (PMC11106156; doi:10.1007/s10151-024-02932-4)
Supplement: Supplementary file 2 — Supplementary file2 (DOCX 17 KB) [file 10151_2024_2932_MOESM2_ESM.docx]

**Supplementary file 2. CHEC-list + assessment instruction**

**CHEC-list - questions**

1. Is the study population clearly described?
2. Are competing alternatives clearly described?
   1. **Question was deleted from original CHEC-list as it did not occur in this study**
3. Is a well-defined research question posed in answerable form
4. Is the economic study design appropriate to the stated objective?
5. Is the chosen time horizon appropriate to include relevant costs and consequences?
6. Is the actual perspective chosen appropriate?
7. Are all important and relevant costs for each alternative identified?
8. Are all costs measured appropriately in physical units?
9. Are costs valued appropriately?
10. Are all important and relevant outcomes for each alternative identified?
    1. **Question was deleted from original CHEC-list as it did not occur in this study**
11. Are all outcomes measured appropriately?
    1. **Question was deleted from original CHEC-list as it did not occur in this study**
12. Are outcomes valued appropriately?
    1. **Question was deleted from original CHEC-list as it did not occur in this study**
13. Is an incremental analysis of costs and outcomes of alternatives performed?
14. Are all future costs and outcomes discounted appropriately?
    1. **Question was deleted from original CHEC-list as it did not occur in this study**
15. Are all important variables, whose values are uncertain, appropriately subjected to sensitivity analysis?
16. Do the conclusions follow from the data reported?
17. Does the study discuss the generalizability of the results to other settings and patient/client groups?
18. Does the article indicate that there is no potential conflict of interest of study cresearcher(s) and funder(s)?
19. Are ethical and distributional issues discussed appropriately?

**Assessment instruction – Value for health economic purposes**

The CHEC-list consists of 19 yes-or-no questions one for each category. This version is adapted from the original CHEC-list. Reporting items that apply only to full health economic analyses are removed from the list. Items are explained below:

1.The relevant clinical characteristics, entry and eligibility criteria, as well as drop-out during follow-up should be stated explicitly.

2.A detailed description should be given of the competing interventions. This should encompass a clear and specific statement of the primary objective of each alternative, as well as relevant factors, such as intensity, duration, and frequency.

- **Question was deleted from original CHEC-list as it did not occur in this study**

3.A research question has to identify clearly the alternatives being compared and the population for which the comparison is made.

4.An appropriate economic study design is a cost of illness design.

5.The period of analysis of the study is the time horizon. This time horizon should always be equal for costs and outcomes if these are combined in a ratio. The time span should be long enough to include all relevant costs and outcomes relating the intervention. Ideally, the follow-up period should be extended till the situation is stabilised with reference to costs and effects.

- Only costs index admission is a ‘no’
- Only AL during index admission is a ‘no’
- Time of costs and time of AL should be equal

6. ‘Perspective’ indicates from which point of view an economic evaluation study is performed. If the study is performed from a societal perspective tick ‘yes’, as all relevant costs and consequences of an interventions and disease are taken into account, if possible. Other narrower perspectives will only include certain components. The authors should motivate why a narrower perspective is valid.

- Societal impact is a ‘yes’
- Only own hospital perspective is a ‘no’
- Broadening perspective to other countries or hospitals is a ‘yes’

7.A full identification of all important and relevant costs should be given in relation to the perspective and the research question.

- ‘Yes’ are all relevant cost units for their specific perspective and research question taken into consideration.

8.The costs should be measured appropriately in physical units. The instrument by which the costs are measured should be valid and clearly stated (e.g. interview, questionnaire, cost-diary).

- Are all costs defined in question 7 measured appropriately.

9.The sources of valuation should be clearly stated for each cost price of every volume parameter and their reference year. The main cost should be calculated based on depleted sources, no tariffs should be used.

- Reference years should be stated, otherwise ‘no’
- Bottom-up otherwise ‘no’

10. A full identification of all important and relevant outcomes should be given in relation to the perspective and the research question.

- **Question was deleted from original CHEC-list as it did not occur in this study**

11. The outcome measurement should result from the outcome identification and this should be straightforward (e.g. if mortality is a main outcome measure this should be taken into account in the analysis). The instrument by which the outcomes are measured should be valid and clearly stated.

- **Question was deleted from original CHEC-list as it did not occur in this study**

12. The method of outcome valuation should be clearly stated. Examples of valuation methods are Discrete Choice Experiments (e.g. Conjoint analysis, Contingent valuation), Direct utility assessment (VAS, TTO, SG, etc.), Indirect utility assessment (HUI, EQ-5D, QWB, etc.), Person trade off, etc.

- **Question was deleted from original CHEC-list as it did not occur in this study**

13. An incremental analysis should examine the additional costs from one intervention over another, ~~compared to the additional outcomes that it delivers.~~ The incremental costs-effectiveness ratio is obtained by dividing the costs differences (C2-C1) by the outcome differences (O2-O1) for the alternatives.

- Comparison with no AL should be clearly stated

14. Discounting is done appropriately if all costs and outcomes are converted to one single year, based on a motivated discount rate.

- **Question was deleted from original CHEC-list as it did not occur in this study**

15. All variables in the analysis are potential candidates for the sensitivity analysis. Only variables that are certain or which have a minimal impact on the study results (based on the preliminary analysis) can be excluded from the sensitivity analysis. Furthermore, a justification should be given over the range of the variables used in the sensitivity analysis.

16. Do the authors interpret their results cautiously and are their conclusions justified by the data.

17. This can be done by being explicit about the viewpoint of analysis and by indicating how particular costs and outcomes vary by location, setting, patient population, care provider, etc.

18. If an external agency finances the study, a statement should explicitly be given about who finances the study to guarantee transparency in the relationship between the sponsor and the researcher. Whenever a potential conflict of interest is possible a declaration should be given of ‘competing interest’.

19. Does the article notes ethical aspects and elaborates on the characteristics of the population experiencing the disease or the intervention (young, old, poor, wealthy) and how this may have distributional implications.
